# Superior Mesenteric Vein Thrombosis in a Patient Treated for Internal Hernia and Right Hemicolectomy: A Case Report and Review of the Literature

**DOI:** 10.7759/cureus.90676

**Published:** 2025-08-21

**Authors:** Nefeli Tatsina, Nikolaos Koliakos, Eleonora Farinella, Andries Ryckx

**Affiliations:** 1 Internal Medicine, Saint-Luc Hospital, Brussels, BEL; 2 Digestive Surgery, Saint-Pierre Hospital, Brussels, BEL; 3 General Surgery, Centre Hospitalier de Luxembourg (CHL), Arlon, BEL

**Keywords:** anticoagulation, internal hernia, right hemicolectomy, roux-en-y gastric bypass, superior mesenteric vein thrombosis

## Abstract

We report a rare case of chronic superior mesenteric vein (SMV) thrombosis (SMVT) secondary to an internal hernia in a 65-year-old man with prior laparoscopic Roux-en-Y gastric bypass. He was evaluated for a colonic polyp suspicious for malignancy. Imaging revealed Petersen’s hernia and chronic SMVT with collateral formation. He underwent laparoscopic hernia reduction with mesenteric defect closure and right hemicolectomy. Postoperatively, he was treated with rivaroxaban. A gastrojejunostomy ulcer caused hematemesis during postoperative recovery; this was managed endoscopically with stopping and bridging of anticoagulation. Follow-up imaging confirmed SMV recanalization. This case highlights the importance of recognizing internal hernia as a rare cause of SMVT. Surgical correction with anticoagulation can result in excellent outcomes.

## Introduction

Internal hernia is a common surgical complication following laparoscopic Roux-en-Y gastric bypass (LRYGB). Both Petersen’s and jejuno-jejunal mesenteric defects can cause small bowel herniation leading to obstruction and eventually strangulation and necrosis [1]. Fortunately, with the current routine closure of mesenteric defects, only 2% of patients present with symptomatic internal herniation post-LRYGB [2,3]. Patients presenting with abdominal pain and a history of LRYGB should be routinely examined with abdominal computed tomography (CT) [4,5]. Typical CT findings considered pathognomonic for the diagnosis of an internal hernia are the swirl sign, small bowel loops behind the superior mesenteric artery, and the presence of the jejuno-jejunostomy in the upper quadrant [6].

However, besides acute presentations, internal hernias can exist chronically and lead to persistent venous congestion, which can progress to superior mesenteric vein (SMV) thrombosis (SMVT). SMVT is a severe but rare complication, reported in less than 1% of Roux-en-Y gastric bypass (RYGB) patients, but often leading to severe additional morbidity and mortality. Besides mechanical causes for SMVT, other diseases leading to a hypercoagulation state (such as cancer) have been described in the presence of diagnosed SMVT.

We present a case of chronic SMVT with imaging of an internal hernia after RYGB in a patient undergoing a combined laparoscopic reduction and closure of internal hernia and right hemicolectomy for colon cancer. This is, to our knowledge, the first published case describing a chronic SMVT with a documented internal hernia after RYGB in a patient with colon cancer.

## Case presentation

A 65-year-old man was seen in the clinic with a diagnosed right colonic polyp. He was treated for arterial hypertension with Coversyl 5 mg/day and for gout with allopurinol 300 mg/day. He was treated for sleep apnea with a continuous positive airway pressure (CPAP) device (six hours/night). His arterial hypertension was well controlled with daily measurements at home. His past surgical history included a RYGB for morbid obesity (BMI 45 kg/m^2^) 10 years before his admission, with a total weight loss of 42 kg and a current BMI of 32 kg/m^2^. He had no history of abdominal pain or obstruction, and during the physical examination, his abdomen was soft and non-tender. There was no history of thromboembolic events. He does not smoke. Laboratory data are shown in Table 1.

**Table 1:** Laboratory data INR: international normalized ratio

Term	Value	Measure	Reference
Hemoglobin	12.5	g/dL	12.1-17.2
White blood cell count (WBC)	4.1	cells x 10^9^/L	4.5-11
Platelet count	3.1	cells x 10^9^/L	1.5-4.5
C-reactive protein (CRP)	2.8	mg/L	<5
Creatinine	1.2	µmol/L	0.7-1.3
Carcinoembryonic antigen (CEA)	2.9	µg/L	<3
D-dimers	700	ng/L	>500
INR	2.6		2.0-3.5

A total colonoscopy detected an ulcerated adenoma suspected of malignancy at the ileocecal valve. A biopsy showed a tubular adenoma with low-grade dysplasia. Contrast-enhanced abdominal CT showed the presence of the reported large polyp. As per protocol, there was an incidental finding of Petersen’s hernia and a diagnosis of a chronic SMVT.

**Figure 1:**
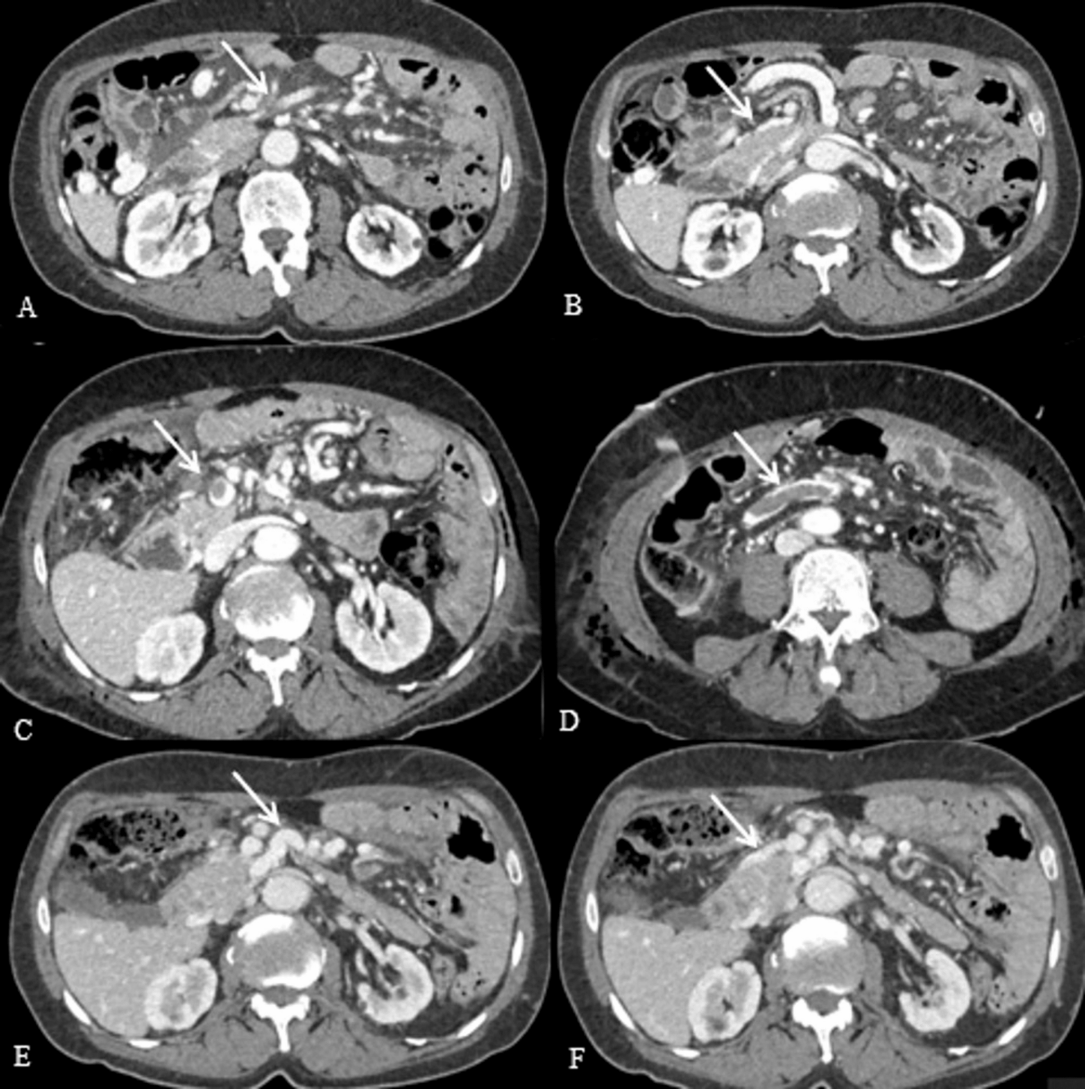
Contrast-enhanced computed tomography Coronal images revealing superior mesenteric vein filling defects, collaterals, and a partially enhanced thrombus before (A, B) and after (C, D) surgery. Three months postoperatively, after anticoagulation treatment, the superior mesenteric vein has been recanalized and no filling defects are visible (E, F).

The thrombosis did not extend into the splenic or portal vein. Portosystemic collateral vessels had been formed at the level of the ileocolic and right colic vein, confirming the chronic aspect of the SMVT (Figures 1A, 1B).

A multidisciplinary meeting was organized, and an elective laparoscopic right hemicolectomy and internal hernia reduction were planned. The patient consented to the procedure.

Intraoperatively, following the reduction of the internal hernia, there were no signs of bowel ischemia or serosal ischemic discoloration; there were visible distended venous mesenteric vessels in the mesocolon. Petersen’s space was sutured by non-absorbable running suture (Prolene 2-0, Ethicon, Raritan, NJ, US).

The patient then underwent a standard laparoscopic right hemicolectomy with no touch technique and a central ligation of the ileocolic artery and the right branch of the middle colic artery at the origin of the SMV. The large mesocolic veins were ligated with the use of the Hem-o-Lok polymer ligating XL^®^ clip (Weck Closure Systems, Research Triangle Park, NC, US). The terminal ileum and transverse colon were transected using a 60 mm laparoscopic stapler (Endo GIA™ Stapler, Medtronic, Dublin, Ireland). Restoration of the bowel continuity was performed with an intracorporeal isoperistaltic semi-mechanical side-to-side ileo-transverse anastomosis, and the stapler orifice was closed with a running V-lock 3-0 suture. The estimated blood loss was 500 mL because of venous congestion in the resected specimen.

The postoperative course was uneventful; the patient had flatus at D1 and was discharged on the fourth postoperative day with rivaroxaban 20 mg PO qDay. No complications were reported. Pathological examination of the right hemicolectomy specimen revealed an in situ adenocarcinoma arising from a 6 cm tubulovillous adenoma. No adjuvant treatment was deemed necessary.

Two weeks following his discharge, he was readmitted to our department due to hematemesis and moderate anemia (hemoglobin (Hb) 9.5 g/dL). Esophagogastroduodenoscopy (EGD) revealed an active-oozing ulcer at the gastrojejunostomy classified as Forrest Ib (GJ). Endoscopic hemostasis with clipping was successfully performed; no transfusion was needed.

Rivaroxaban was stopped at admission and restarted three days after bleeding control; the patient was bridged with prophylactic low-molecular-weight heparin (LMWH). The HAS-BLED score before the bleeding was calculated at 2 and after at 3, leading to a risk of major bleeding of 4.1% vs. 5.8% [7]. Given the high risk of recurrence of SMVT shown by his past history and the mechanical control of the bleeding with hemostatic clips lowering significantly the risk of recurrence and the low HAS-BLED score, rivaroxaban was restarted on day 4. He was also discharged on a proton-pump inhibitor at a high dose (80 mg/day).

The patient continued treatment with rivaroxaban for three months, and he received a comprehensive evaluation of a pro-thrombotic state (analysis of antiphospholipid syndrome, protein S and C deficiency, antithrombin III levels, and factor V and Leiden mutation) without revealing any pathology. Follow-up examinations with CT confirmed the resolution of the SMVT (Figures 1E, 1F). This report complies with the Declaration of Helsinki and CARE guidelines; written informed consent was obtained from the patient. 

## Discussion

Routine non-closure of mesenteric defects after LRYGB is related to increased risk of a postoperative internal hernia [2]. Despite the fact that routine closure reduces the incidence of internal hernia, it is not without associated risks. Mesenteric bleeding can lead to hematoma, edema, and other complications [4,5]. Furthermore, a meta-analysis showed higher rates of early small bowel obstruction following mesenteric defect closure mainly due to possible kinking of the jejunojejunostomy [1]. Despite obvious benefits, routine closure of mesenteric defects is not performed in about 5% of LRYGB due to intraoperative difficulties or an estimated increased risk of complications [2].

Internal hernia leads to entrapment, kinking, or swirling of bowel and mesentery, which in turn causes mesenteric vessel congestion and eventually venous thrombosis. A more pronounced mechanical congestion can lead to a more extended venous thrombosis and thrombosis extending to the SMV and subsequently to the portal vein [8].

**Figure 2:**
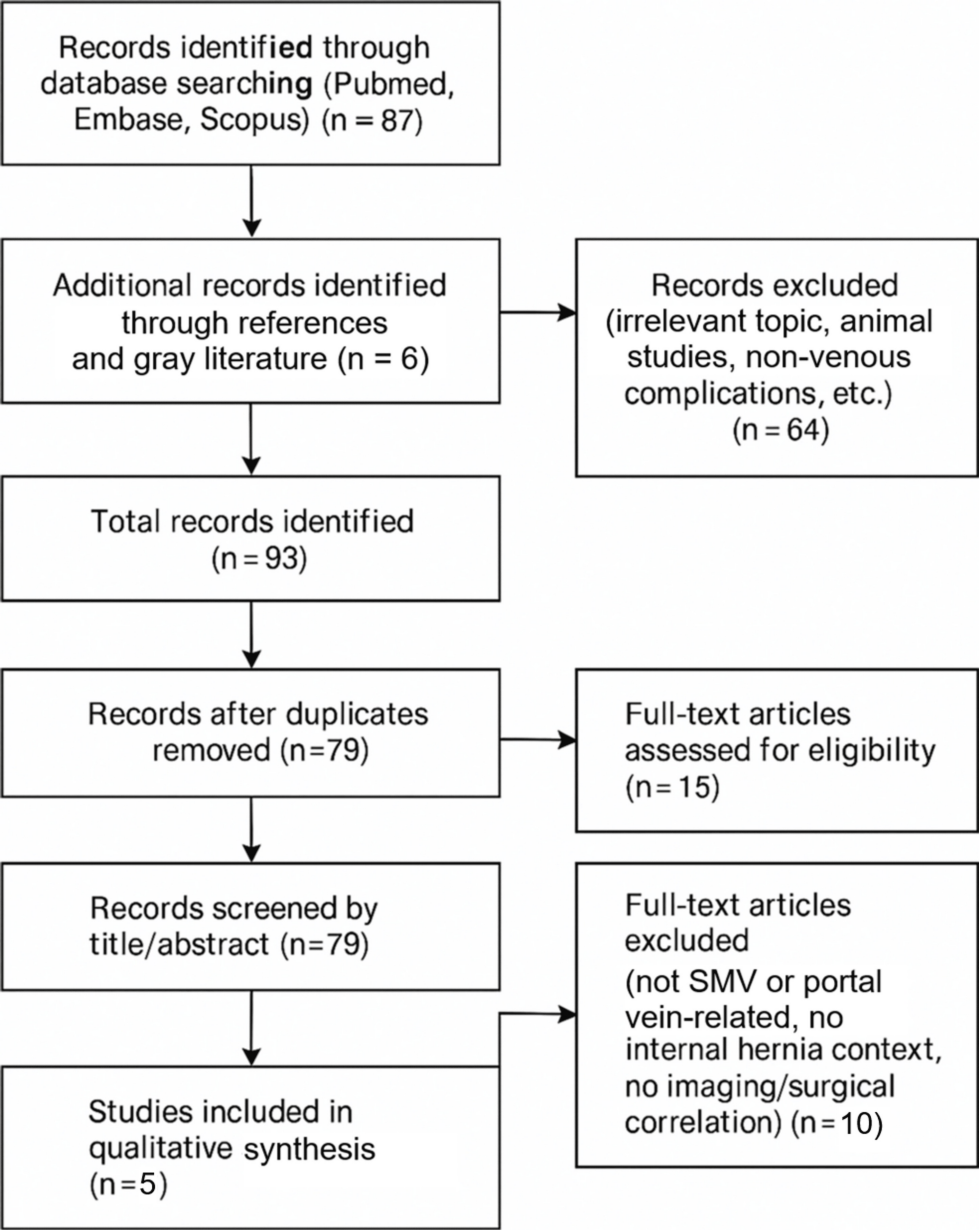
PRISMA diagram PRISMA: Preferred Reporting Items for Systematic reviews and Meta-Analyses; SMV: superior mesenteric vein

A structured literature search (PubMed, Scopus, and Google Scholar; 1990-2025) identified five published cases linking internal hernias to mesenteric venous events, including SMVT, porto-mesenteric thrombosis, or outflow obstruction. The Preferred Reporting Items for Systematic reviews and Meta-Analyses (PRISMA) diagram outlines the search and selection process (Figure 2).

The inclusion and exclusion criteria are summarized in Figure 2. These findings reinforce a pathophysiological role for mechanical venous compromise in internal hernias, which may present with ischemia, thrombosis, or variceal bleeding depending on the degree and duration of obstruction. Articles are summarized in Table 2.

**Table 2:** Published cases of internal hernia after RYGB with associated mesenteric venous events (1990–2025) RYGB: Roux-en-Y gastric bypass; SMVT: superior mesenteric vein thrombosis; CT: computed tomography; SMV: superior mesenteric vein

Author (year)	Hernia type	Procedure history	Venous event	Portal vein involved	Imaging/operative confirmation	Outcome
Kimball et al. (2012) [9]	Petersen's (RYGB)	RYGB	Porto-mesenteric thrombosis	Yes	CT confirmed	Bowel resection
Fabozzi et al. (2014) [10]	Petersen's (RYGB)	RYGB	SMV and portal vein thrombosis	Yes	CT + operative findings	Bowel necrosis, resection
Shi et al. (2025) [11]	Petersen's (RYGB)	RYGB	SMV stenosis → ectopic variceal bleeding	Functional only	CT + endoscopy	Hemostasis + surgical correction
Clements et al. (2025) [12]	Petersen's (RYGB)	RYGB	Isolated SMVT	No	CT confirmed	Anticoagulation, full recovery
Dar et al. (2020) [13]	Transmesenteric (suspected)	RYGB	Portal vein and SMVT (unspecified)	Yes	Radiologic description	Surgical reduction, partial recovery

If the entrapment persists, bowel necrosis requiring urgent surgical exploration can ensue, leading to high morbidity, with a reported estimated mortality of 30% [4]. Radiologically important differences can be made between acute and chronic SMVT (Table 3) [7].

**Table 3:** Radiological features of acute vs. chronic MVT MVT: mesenteric vein thrombosis; SMV: superior mesenteric vein; CT: computed tomography

Feature	Acute MVT	Chronic MVT
SMV appearance	Dilated, hyperdense (on unenhanced CT), non-enhancing intraluminal filling defect	SMV may be small, narrowed, or occluded; may show collateral vessels
Bowel wall	Wall thickening, edema, possible enhancement or lack thereof (ischemia)	Normal or slightly thickened bowel; fibrosis possible
Mesenteric fat	Stranding, edema, and inflammatory changes	Less or no fat stranding; may show fibrotic changes
Bowel loops	Distended, fluid-filled, hypo- or non-enhancing loops (if ischemia present); may see pneumatosis or portal venous gas in severe cases	Usually normal; bowel adapts unless chronic ischemia/fibrosis has occurred
Venous collaterals	Typically absent in early stages	Present, indicating long-standing venous outflow obstruction
Ascites	Possible moderate ascites (serosanguinous)	Often absent or minimal
Portal vein/splenic vein	May also show acute thrombosis if extensive	May have cavernous transformation (network of small collateral veins)
Enhancement pattern	Non-enhancing thrombus within enhanced vein	Organized thrombus may have partial enhancement or calcification
Time course	Sudden onset of symptoms; radiologic findings correlate with acute vascular compromise	Often incidental or discovered due to chronic abdominal complaints or workup for portal hypertension

Mesenteric vein thrombosis represents approximately 25% of reported splanchnic vein thrombotic events [14]. Current management of venous-related ischemia consists of systemic anticoagulation as per guidelines [15]. Anticoagulation with a continuous infusion of unfractionated or LMWH is the recommended emergency medical treatment, preventing thrombus extension without significantly increasing the risk of bleeding. The rate of minor or major bleeding during anticoagulation treatment with rivaroxaban has been described at 2%. However, prolonged treatment is advised since the risk of splanchnic vein thrombosis, pulmonary embolism, or cerebral vein thrombosis during treatment remains elevated [10]. Anticoagulation is considered the cornerstone of medical treatment, preventing thrombus extension without increasing the risk of any bleeding [16,17]. SMV recanalization following internal hernia reduction requires, on average, a few days to three months. Specifically, when treating isolated mesenteric vein thrombosis, anticoagulants increase the possibility of recanalization dramatically [18].

The bleeding lesion was classified as Forrest Ib (oozing) and treated endoscopically, followed by high-dose intravenous proton pump inhibitor (PPI) in line with European Society of Gastrointestinal Endoscopy (ESGE) 2021 guidelines, which support hemostasis for non-variceal upper gastrointestinal (GI) bleeding and recognize Forrest Ib as high-risk [19]. Regarding anticoagulation resumption, the 2022 American College of Gastroenterology (ACG)-Canadian Association of Gastroenterology (CAG) joint guideline acknowledges the lack of definitive timing data for direct oral anticoagulants (DOACs), instead recommending individualized decisions based on thrombotic vs. rebleeding risk [20]. Observational studies suggest that early resumption (within three days) may reduce thrombotic events without consistently increasing rebleeding [21,22]. Notably, one analysis paradoxically reported higher rebleeding rates when DOACs were resumed after day 3, potentially due to selection bias and bridging with LMWH [23]. In this high-thrombotic-risk patient with chronic SMVT, our multidisciplinary team (MDT) opted for temporary interruption, prophylactic LMWH bridging, and rivaroxaban resumption on day 3, balancing bleeding stability against thrombosis prevention.

The accumulating evidence underscores a clinically significant message: mechanical causes such as internal hernias can play a direct pathophysiological role in the development of SMVT, and prompt surgical correction combined with anticoagulation can result in favorable outcomes, including bowel preservation and vascular recanalization. Nonetheless, a key limitation across reported cases-and in our own experience-is the inability to definitively establish temporal causality. It remains unclear whether SMVT arises primarily as a consequence of mechanical obstruction and venous outflow compromise due to the hernia or whether a pre-existing thrombosis leads to altered mesenteric dynamics that promote internal herniation. In most clinical scenarios, including those with chronic or recurrent presentations, the interplay between mechanical and thrombotic processes is likely bidirectional and dynamic.

This diagnostic ambiguity reinforces the need for an individualized, case-by-case approach to management. Surgical exploration may be necessary when ischemia or entrapment is suspected, particularly when radiologic signs suggest vascular compromise. At the same time, anticoagulation remains the cornerstone of therapy in confirmed or suspected SMVT, both to prevent thrombus propagation and to facilitate venous recanalization. Decisions regarding timing, intensity, and duration of anticoagulation-especially in the setting of recent surgery or GI bleeding-require careful multidisciplinary deliberation. Ultimately, recognizing internal hernia as a potentially reversible mechanical cause of SMVT highlights the value of integrated surgical and medical management in optimizing outcomes in these complex cases.

## Conclusions

We are the first to describe a case of concomitant internal herniation after RYGB with documented chronic SMVT and colon cancer treated by surgery and anticoagulation. Guidelines on treatment are scarce and do not take into account mechanical causes such as internal hernia as a possible precursor to SMVT. However, this case shows that a combined surgical treatment of both pathologies in selected patients with added postoperative anticoagulation can lead to excellent results and recanalization of the SMV.

Routine anticoagulation should be carefully considered, given the added risk of bleeding as shown in our case, and score systems are advised to estimate bleeding risk. A specific registry to monitor these kinds of severe complications is valuable and could lead to more insights and possibilities to draw clinically significant conclusions.
